# Evidence for tankyrases as antineoplastic targets in lung cancer

**DOI:** 10.1186/1471-2407-13-211

**Published:** 2013-04-28

**Authors:** Alexander M Busch, Kevin C Johnson, Radu V Stan, Aarti Sanglikar, Yashi Ahmed, Ethan Dmitrovsky, Sarah J Freemantle

**Affiliations:** 1 From the Department of Pharmacology and Toxicology, Geisel School of Medicine at Dartmouth, Hanover, NH, 03755, USA; 2 Heart and Vascular Research Center, Geisel School of Medicine at Dartmouth, Hanover, NH, 03755, USA; 3 Department of Pathology, Geisel School of Medicine at Dartmouth, Hanover, NH, 03755, USA; 4 Department of Microbiology and Immunology, Geisel School of Medicine at Dartmouth, Hanover, NH, 03755, USA; 5 Center for Comparative Medicine and Research, Geisel School of Medicine at Dartmouth, Hanover, NH, 03755, USA; 6 Department of Genetics, Geisel School of Medicine at Dartmouth, Hanover, NH, 03755, USA; 7 Norris Cotton Cancer Center, Geisel School of Medicine at Dartmouth, Hanover, NH, 03755, USA; 8 Department of Medicine, Geisel School of Medicine at Dartmouth, Hanover, NH, 03755, USA

**Keywords:** Lung cancer, Wnt pathway, Tankyrase inhibitors, TNKS, TNKS2

## Abstract

**Background:**

New pharmacologic targets are urgently needed to treat or prevent lung cancer, the most common cause of cancer death for men and women. This study identified one such target. This is the canonical Wnt signaling pathway, which is deregulated in cancers, including those lacking *adenomatous polyposis coli* or *β-catenin* mutations. Two poly-ADP-ribose polymerase (PARP) enzymes regulate canonical Wnt activity: tankyrase (TNKS) 1 and TNKS2. These enzymes poly-ADP-ribosylate (PARsylate) and destabilize axin, a key component of the β-catenin phosphorylation complex.

**Methods:**

This study used comprehensive gene profiles to uncover deregulation of the Wnt pathway in murine transgenic and human lung cancers, relative to normal lung. Antineoplastic consequences of genetic and pharmacologic targeting of TNKS in murine and human lung cancer cell lines were explored, and validated *in vivo* in mice by implantation of murine transgenic lung cancer cells engineered with reduced TNKS expression relative to controls.

**Results:**

Microarray analyses comparing Wnt pathway members in malignant versus normal tissues of a murine transgenic cyclin E lung cancer model revealed deregulation of Wnt pathway components, including TNKS1 and TNKS2. Real-time PCR assays independently confirmed these results in paired normal-malignant murine and human lung tissues. Individual treatments of a panel of human and murine lung cancer cell lines with the TNKS inhibitors XAV939 and IWR-1 dose-dependently repressed cell growth and increased cellular axin 1 and tankyrase levels. These inhibitors also repressed expression of a Wnt-responsive luciferase construct, implicating the Wnt pathway in conferring these antineoplastic effects. Individual or combined knockdown of TNKS1 and TNKS2 with siRNAs or shRNAs reduced lung cancer cell growth, stabilized axin, and repressed tumor formation in murine xenograft and syngeneic lung cancer models.

**Conclusions:**

Findings reported here uncovered deregulation of specific components of the Wnt pathway in both human and murine lung cancer models. Repressing TNKS activity through either genetic or pharmacological approaches antagonized canonical Wnt signaling, reduced murine and human lung cancer cell line growth, and decreased tumor formation in mouse models. Taken together, these findings implicate the use of TNKS inhibitors to target the Wnt pathway to combat lung cancer.

## Background

Lung cancer is the leading cause of cancer mortality for men and women [1,2]. Despite smoking prevention and cessation programs [3] and advances in early detection [4], the 5-year survival rate for lung cancer is only 16% with current therapies [1]. Although lung cancer incidence rates have recently declined in the United States [1], more lung cancer is now diagnosed when considered together in former- and never-smokers than in current smokers [5]. Thus, even if all of the national anti-smoking campaign goals are met, lung cancer will remain a major public health problem for decades. New ways to treat or prevent lung cancer are therefore needed.

One potential therapeutic target for lung cancer is the Wnt signaling pathway [6-9]. The canonical Wnt signaling pathway in mammals consists of a family of secreted lipid-modified Wnt protein ligands that bind to a family of 7-pass transmembrane Frizzled (Fzd) receptors, as reviewed [10]. In brief, in the absence of ligand, glycogen synthase kinase-3 (GSK3), in complex with axin and adenomatous polyposis coli (APC), constitutively phosphorylates β-catenin, the primary Wnt signaling effector, targeting it for ubiquitination and proteasomal destruction. Ligand binding engages a pathway involving Dishevelled (Dvl) that inhibits GSK3, allowing β-catenin to accumulate in a hypophosphorylated form. This stabilized form of β-catenin can translocate to the nucleus, where it activates target gene transcription by complexing with T cell factor (TCF) and lymphoid enhancer-binding factor (LEF). In addition to key mediators of embryonic development, these target genes include critical growth-regulators such as *myc* and *cyclin D1*[11,12].

Aberrant Wnt signaling due to mutations in *β-catenin* or *APC* drives deregulated growth in both familial [13] and non-hereditary colorectal cancers [14,15]. However, non-small cell lung cancers (NSCLC), the most common type of lung cancer, rarely harbor *APC* or *β-catenin* mutations [16]. Rather, aberrant Wnt activity in lung cancer is linked to increased expression of upstream Wnt signaling effectors such as Dvl [17] or decreased expression of Wnt antagonists such as Wnt-inhibitory factor 1 (Wif-1) [18,19].

Effective pharmacological inhibitors of the Wnt pathway have only recently become available. Screens for small-molecule antagonists of the Wnt pathway [20,21] found two enzymes to be key mediators of Wnt signaling. These are poly-ADP-ribose polymerase (PARP) enzymes, tankyrase (TNKS) 1 and TNKS2, which attach poly-ADP-ribose (PAR) onto substrate proteins. Their roles in regulating telomerase function [22] and mitotic spindle formation [23,24] are known, but their role in PARsylating axin so as to maintain the optimal level for canonical Wnt signaling has only recently been recognized. The compounds identified in these screens, XAV939 [20], IWR-1 exo, and IWR-1 endo [21], act by specifically inhibiting the PARP activity of TNKS1 and TNKS2 [25,26]. IWR-exo is a stereoisomer of IWR-1 endo with ~14-fold lower EC_50_[21]. PARP inhibition is a tractable pharmacological target *in vivo*, as antagonists of other PARP homologs exert antineoplastic responses in breast and ovarian cancer [27,28], as reviewed, [29].

This study explored the hypothesis that inhibition of TNKS by pharmacological or genetic means would inhibit lung cancer growth *in vitro* and *in vivo* in clinically-relevant transgenic mouse models of lung cancer that were previously developed, as reviewed [30]. Using comprehensive microarray analyses, we found that TNKS were overexpressed in murine lung cancers relative to adjacent normal lung tissues. These results were confirmed by semi-quantitative real-time polymerase chain reaction (qPCR) assays. Individual treatments of a well-characterized panel of human and murine lung cancer cell lines with the TNKS inhibitors XAV939 or IWR-1 inhibited cell growth, reduced the activation of a Wnt-responsive lentiviral luciferase construct, and stabilized protein levels of axin and both TNKS. Genetic inhibition of TNKS was independently achieved by use of siRNA or shRNA-mediated knockdown in lung cancer cells. This resulted in axin stabilization, marked growth inhibition, and repressed lung cancer formation in murine xenograft and transgenic syngeneic lung cancer models. Taken together, the findings presented here uncover TNKS as new antineoplastic lung cancer targets.

## Methods

### Murine transgenic lung tissues

We previously described clinically-relevant cyclin E-transgenic mouse lines that develop pulmonary pre-malignant lesions and lung adenocarcinomas [31]. For microarray analyses, adenocarcinomas and adjacent histopathologically normal lung tissues were each harvested from age- and sex-matched mice and immediately placed in RNAlater (Qiagen, Valencia CA). These specimens were isolated from human surfactant protein C (SP-C)-driven wild-type human cyclin E-transgenic mice (as previously described [31]). Normal non-transgenic lung tissue was harvested from age-and sex-matched FVB mice (NCI Frederick National Laboratory, Frederick MD). For qPCR analyses, malignant and adjacent normal lung tissues were isolated from additional transgenic mice of both wild-type and proteasome-degradation resistant human cyclin E-transgenic lines and snap frozen in liquid nitrogen.

### Gene expression microarray analyses

Total RNA was isolated with TRIzol RNA isolation reagent (Life Technologies, Carlsbad CA). GeneChip Mouse Genome 430 2.0 Arrays were purchased (Affymetrix, Santa Clara CA), with 11-probe sets covering 39,000 transcripts within the mouse genome. Hybridizations were performed according to Affymetrix guidelines at the Dartmouth College Microarray Shared Resource using an Affymetrix GeneChip Workstation. Biotin-labelled cRNA was generated from 5 μg of total RNA and hybridized to the Mouse Genome 430 2.0 chip. A total of 12 hybridizations were performed comprising 12 independent biologic samples organized into three groups of four. Raw data from each hybridization was normalized by Robust Multichip Average (RMA), background corrected, and filtered for presentation using GeneSifter software (Geospiza Inc., Seattle WA). The remaining probe sets were then analyzed by GeneSifter software for species involved in the Wnt pathway. Raw and RMA data is available from the NCBI Gene Expression Omnibus (GEO) with accession number GSE45744.

### Paired human-malignant lung tissues

A tissue bank accrued from consecutive cases over 8 years at Dartmouth-Hitchcock Medical Center containing paired human normal and malignant lung tissues was described [32]. Dartmouth’s Institutional Review Board (IRB) reviewed and approved the acquisition and analyses of these tissues.

### Semiquantitative real time RT-PCR assays

Total mRNA was isolated using the RNeasy kit with on-column DNAse digestion (Qiagen). RT was performed with the High Capacity cDNA RT Kit (Applied Biosystems, Foster City CA) and a Peltier Thermal Cycler (MJ Research, Waltham MA). The qPCR assays were performed using iTaq Fast SYBR Green Supermix with ROX (Bio-Rad Laboratories, Hercules CA) and the 7500 Fast Real-Time PCR System (Applied Biosystems). All assays were performed in triplicate. Primers sequences are presented in Additional file 1: Figure S1.

### Cell culture

Murine lung cancer cell lines studied included ED1, ED2, and ED1L (derived from a single-cell subclone of ED1), which were each previously described [32,33]. The C-10 immortalized murine bronchial epithelial cell line, BEAS-2B immortalized human bronchial epithelial cell line, NCI-H522, Hop62, and A549 human lung cancer cell lines, and the 293T human embryonic kidney cell line were each purchased (ATCC, Manassas VA). All cell lines except BEAS-2B and 293T cells were cultured in RPMI 1640 medium (Corning, Manassas VA) supplemented with 10% fetal bovine serum (FBS; Thermo Fisher Scientific, Waltham MA) at 37°C in a 5% CO_2_ humidity-controlled incubator. BEAS-2B cells were cultured in serum-free LHC-8 medium (Life Technologies) supplemented with 0.1% epinephrine. The 293T cell line was cultured in high glucose DMEM (Life Technologies) supplemented with 10% FBS and 4 mM L-glutamine (Life Technologies).

### Reagents

TNKS inhibitors XAV939 [20], IWR-1 endo, and IWR-1 exo [21] were purchased (Cayman Chemical, Ann Arbor MI) and dissolved in dimethyl sulfoxide (DMSO; Sigma-Aldrich, St. Louis MO). Recombinant murine Wnt3a ligand was purchased (R&D Systems, Minneapolis MN) and dissolved in 1% bovine serum albumin (BSA; Sigma-Aldrich) in phosphate buffered saline (PBS; Corning).

### Proliferation, clonogenicity, and washout studies

For cell proliferation assays, ED1 (2 × 10^3^), ED1L (2 × 10^3^), ED2 (5 × 10^3^), C-10 (5 × 10^3^), BEAS-2B (5 × 10^3^), H522 (5 × 10^3^), and A549 (5 × 10^3^) were individually plated in growth medium in triplicate in individual wells of 12-well tissue culture plates (Corning) 24 hours before drug or vehicle treatments. Cell viability was measured 72 hours following these treatments using the CellTiter-Glo (Promega, Madison WI) luminescent cell viability kit and a TD-20/20 Luminometer (Turner Designs, Sunnyvale CA).

For clonal growth assays, ED1 cells were plated at a density of 200 cells per well in 6-well tissue culture plates (Corning) in triplicate 24 hours before drug or vehicle treatments. Colonies were stained after 7 days with DiffQuick (IMEB Inc, San Marcos CA) and counted using a Col Count instrument (Oxford Optronix, Oxford UK).

For washout studies, ED1 (3 × 10^4^) and A549 (7.5 × 10^4^) were independently plated in 10 cm tissue culture plates (Corning) in complete growth medium and individually treated 24 hours later with vehicle, XAV939, IWR-1 endo, or IWR-1 exo at 10 μM dose. Following 3 days of culture in drug, plates were trypsinized and replated at equal densities into 12-well plates in complete medium, as described for cell proliferation assays above. Cells were treated 6 hours later with vehicle, for control and washout wells, or the respective drug at 10 μM to maintain continuous treatment. Cell viability was assessed after 24 hours and 72 hours of treatment by CellTiter-Glo.

### Immunoblot analyses

Cells were lysed in a modified radioimmune precipitation buffer, as before [34]. Protein concentrations were assayed using the BCA Protein Assay Reagent (Thermo Fisher Scientific). Twenty-five micrograms of protein were size-fractionated using 4-15% gradient sodium dodecyl sulfate-polyacrylamide gel electrophoresis (SDS-PAGE) ReadyGels (Bio-Rad Laboratories) before electroblotting onto nictrocellulose membranes. Membranes were blocked with 5% nonfat milk in 0.1% Tween 20 (Sigma-Aldrich) tris-buffered saline (TBST), which was also the antibody diluent, except in the case of the activated β-catenin antibody (ABC), which was diluted in 1% milk TBST as in prior work [6,35]. Antibodies and dilutions used are displayed in Additional file 1: Figure S1. Primary antibodies were detected with horseradish peroxidase-conjugated species-appropriate secondary antibodies (Santa Cruz Biotechnology, Santa Cruz CA and GE Healthcare Bio-Sciences Corp, Piscataway NJ) and visualized with the ECL Prime electrochemiluminescent detection reagent (GE Healthcare) and radiographic film.

### Transient transfection assays

For siRNA knockdown experiments, pairs of independent double-stranded siRNAs were purchased (Integrated DNA Technologies Inc., Coralville IA) that each targeted human or mouse TNKS1 or TNKS2. SiRNA sequences are presented in Additional file 1: Figure S1. A non-targeting scrambled siRNA was used as the control. ED1 (1 × 10^4^), ED2 (1.5 × 10^4^), A549 and (5 × 10^4^), Hop62 (5 × 10^4^) cells were independently plated in triplicate on 6-well tissue culture plates 24 hours before transfection. Transient transfections of each respective siRNA were accomplished with Lipofectamine 2000 reagent (Invitrogen) according to the manufacturer’s protocol. Total RNA was collected and analyzed as described above to verify these knockdowns at 24 hours post-transfection, with cell growth assessed 72 hours post-transfection by CellTiter-Glo assay, as already described. Comparisons were made to cells transfected with the non-targeting scrambled control siRNA.

### Lentivirus production, stable infections, and luciferase assays

The 7TFP derivation of the pSuperTOPFlash vector and the EβP constitutively active β-catenin vector [36] were purchased (plasmids 24308 and 24313, respectively; Addgene, Cambridge MA). Two independent shRNA constructs targeting murine TNKS1 in a G418-selectable backbone, pLKO.1-CMV-Neo, and TNKS2 in a puromycin-selectable backbone, TRC2, were purchased, as well as scrambled controls in matched selectable backbones (Sigma-Aldrich). The sequences of these hairpin constructs are shown in Additional file 1: Figure S1.

Lentiviruses were generated with an optimized system [37] consisting of the transfer vector of interest and packaging plasmids pCMV-dR8.2 (plasmid 8455; Addgene) and pMD2.G (plasmid 12259; Addgene). These vectors were transfected into 293T cells using TransIT-LT1 transfection reagent (Mirus Bio, Madison WI) according to the manufacturer’s protocol. Lentiviral titers supplemented with 1% BSA were collected after 24 hours and used to infect murine ED1 lung cancer cells in the presence of 4ug/mL polybrene (Sigma-Aldrich). ED1 cells infected with 7TFP, EβP, or TNKS2 shRNA vectors were selected with 2.5 μg/mL puromycin (Life Technologies). ED1 cells infected with the TNKS1 shRNA vectors were selected with 1.5 mg/mL G418 sulfate (Corning). ED1 cells transduced with the combined controls or combined shRNAs received simultaneous drug selection at the above concentrations.

For luciferase assays, ED1 cells infected with the 7TFP vector (1 × 10^4^) were plated 24 hours before drug or vehicle treatments in triplicate in 12-well tissue culture plates. Treatments were with TNKS inhibitors or vehicle combined with a canonical Wnt activator or control (20 mM LiCl controlled by 20 mM NaCl or 25 ng/mL murine recombinant Wnt3a controlled by 1% BSA/PBS). Cells were lysed 16 hours after these treatments using the Reporter Lysis Buffer for the Luciferase Assay system (Promega). Luciferase activity was measured and normalized to protein concentrations.

### *In vivo* tumorigenicity studies

The described animal protocols were reviewed and approved by Dartmouth’s Institutional Animal Care and Use Committee (IACUC). For both experiments, ED1 cells were infected with either both shRNA vector controls (dual shCTRL) or TNKS1 and TNKS2 combined knockdown (dual shTNKS) and selected, as described above. For xenograft studies, 1 × 10^6^ indicated cells were resuspended in 200μL of Growth Factor Reduced Matrigel (BD Biosciences, San Jose CA) and injected into the left flanks of 8 week old female NCr Nu/Nu athymic mice (NCI Frederick National Laboratory, Frederick MD). There were 10 mice in the dual control arm and 10 mice in the dual knockdown arm. Tumor diameters were measured twice weekly with vernier calipers by an investigator blinded as to the cell lines under analysis, and mice were sacrificed when mean tumor diameter reached 15 mm or when mice became moribund or cachexic, whichever arose first. Tumor volume was calculated as π/6 * Length * Width^2^, where width was defined as the smaller of the cranial/caudal diameter or dorsal/ventral diameter [38].

For the syngeneic study, 1 × 10^6^ indicated cells were resuspended in 200μL PBS and injected into the tail veins of 8-week-old female FVB mice (NCI Frederick). There were 3 mice in the dual control arm and 5 mice in the dual shRNA arm. Mice were sacrificed 4 weeks post-injection and lung tissues were formalin-fixed, paraffin-embedded, and sectioned for histopathology, as before [31]. Hematoxylin and eosin staining was used and a pathologist blinded as to the treatment arms scored for lung tumor formation, as in prior work [39,40].

### Statistics

Data shown represent at least three independent replicate experiments done in triplicate for the *in vitro* studies. Error bars indicate mean +/- standard deviation (SD), except in the case of the xenograft study, where error bars indicate mean +/- standard error of the mean (SEM). Statistical significance was determined by two-tailed *t*-test assuming unequal population variances in GraphPad InStat or Prism (GraphPad Software Inc, LaJolla CA) with significance set at *P* ≤ 0.05, except in the case of the syngeneic study, when a one-tailed *t*-test was used, and the xenograft study, where ANOVA was used to compare the growth curves and Kaplan-Meier analysis was used to compare survival to sacrificial endpoint. Multiple comparison in the proliferation studies was handled with ANOVA followed by Dunnett’s post-test. Microarray data were analyzed with the GeneSifter analysis suite using the embedded ANOVA function, with significance at *P* ≤ 0.05.

## Results

### The Wnt pathway and TNKS 1 and 2 are deregulated in lung cancer

We previously reported a cyclin E-expressing murine transgenic lung cancer model [31] that recapitulated frequent features of human lung carcinogenesis [32,39-41]. We first asked whether this model deregulated the Wnt pathway, as occurs in human NSCLC [9,16-19]. Comprehensive microarray analyses compared non-transgenic murine lung, transgenic murine normal lung, and transgenic murine lung cancer. These analyses established Euclidean hierarchical clustering of each tissue by Wnt family member expression, with similar expression levels of these species detected in murine non-transgenic lung and transgenic normal lung tissue, but a different expression pattern in transgenic lung cancers (microarray data available at NCBI’s Gene Expression Omnibus, accession number GSE45744).

**Figure 1:**
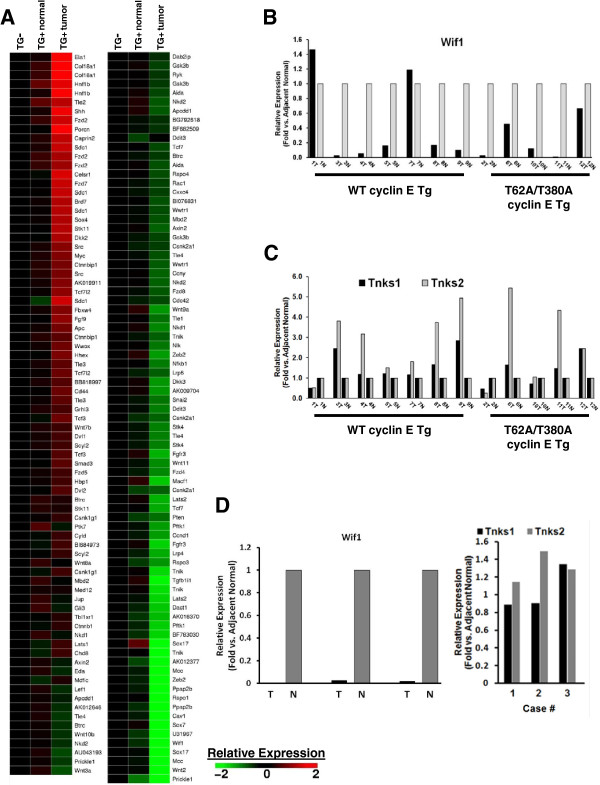
**Wnt pathway deregulation in murine and human NSCLC.** Comprehensive gene expression microarrays and qPCR assays reveal deregulation of specific components of the Wnt signaling pathway in murine and human NSCLC. (**A**)161 probes on the array representing 117 unique genes defined by Gene Ontology under the classification *Wnt receptor signaling pathway* are significantly (ANOVA, *P* ≤ 0.05) over- or under-expressed in murine cyclin-E transgenic lung cancers as compared to adjacent normal or non-transgenic mouse lung. (**B**) The qPCR-based assays of Wif1 expression levels in a panel of paired malignant (samples labeled T) and normal (samples labeled N) lung tissues from 12 transgenic cyclin E mice, both wild type (WT) and proteasome-degradation resistant (T62A/T380A). (**C**) The qPCR-based measurements of TNKS1 and TNKS2 expression levels in the same panel of paired malignant and normal murine lung tissues. (**D**) The qPCR-based measurement of Wif1 (left panel) and TNKS1 and TNKS2 (right panel) expression levels in three human lung adenocarcinoma tumor samples versus adjacent normal lung tissue.

Five hundred probes on the array represent 258 unique genes that are functionally annotated in Gene Ontology (http://www.geneontology.org) as members of the *Wnt receptor signaling pathway*. In our focused analysis of this pathway, 161 probes representing 117 unique genes (32% and 45% of the totals, respectively) were significantly (*P* ≤ 0.05 by ANOVA) up- or downregulated in comparison to the murine non-transgenic or adjacent transgenic normal lung controls (Figure 1A). Among the overexpressed mRNAs were: *porcupine (PORCN),* encoding an O-acyltransferase responsible for lipid-modifying Wnt ligands prior to secretion [42]; *FZD2*, encoding a Wnt/Ca^2+^ signaling-related Frizzled receptor [43]; and *MYC*. Repressed genes included those coding for Wnt antagonists such as WIF1 (discussed above) and PRICKLE1, a putative tumor suppressor that represses the Wnt pathway by destabilizing Dvl [44]. TNKS 1 and 2 were found to be moderately overexpressed, at 1.30-fold and 1.43-fold expression respectively, in comparison with transgenic normal adjacent lung.

The microarray results were independently validated by qPCR assays in murine lung cancers and their adjacent normal lung tissues. In a panel of 12 paired normal and malignant lung tissues derived from 7 wild-type cyclin E transgenes and 5 proteasome-degradation resistant cyclin E transgenes, 10 showed repression of WIF1 in the lung cancers versus adjacent normal lung tissues (Figure 1B). This result is consistent with prior published work from human NSCLC cases [18,19]. Analysis of TNKS 1 and TNKS2 expression in the same panel showed overexpression of TNKS1 in 6 of 12 tumors and TNKS2 in 9 of 12 tumors, relative to adjacent normal lung tissues (Figure 1C). The qPCR analysis of WIF1 and TNKS expression in three cases of human lung adenocarcinoma revealed the expected repression of WIF1 and moderate overexpression of TNKS2 (as compared to adjacent normal lung tissues) in all examined cases, as well as for TNKS1 in 1 of 3 cases (Figure 1D).

### Antiproliferative effects of TNKS inhibitors in lung cancer cells

**Figure 2:**
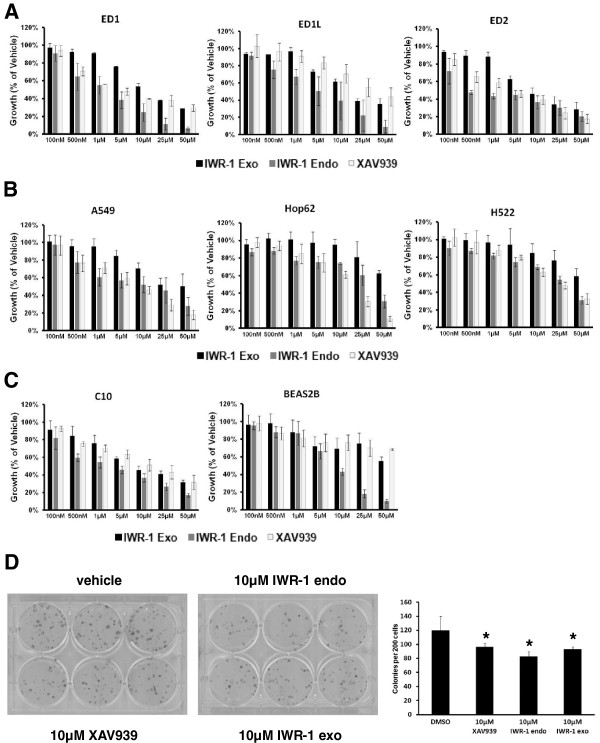
**Antineoplastic effects of TNKS inhibitors *in vitro.*** Treatments with TNKS inhibitors XAV939, IWR-1 endo, and IWR-1 exo exerted antineoplastic effects against NSCLC cell lines *in vitro*, as compared to vehicle controls. (**A**) Cell proliferation dose-response curves for the three TNKS inhibitors against ED1 (left panel), ED1L (middle panel), and ED2 (right panel) murine lung cancer cell lines as compared to vehicle control are shown, as measured by luminescent cell viability assay after 3 days. (**B**) Cell proliferation dose-response relationships for the three TNKS inhibitors against A549 (left panel), Hop62 (middle panel), and H522 human NSCLC cell lines as compared to vehicle control are shown, using the same luminescent cell viability assay and 3 day time frame. (**C**) Dose-response curves show antiproliferative effects of the three TNKS inhibitors against the immortalized bronchial epithelial cell lines C-10 (murine) and BEAS-2B (human) versus vehicle control. (**D**) Ten-day colony formation is shown for the ED1 cell line following individual treatment with 10 μM of each TNKS inhibitor or vehicle control (left two images), and quantified (right panel). Error bars represent standard deviations of three experiments in triplicate. (* *p* ≤ 0.05).

Having established deregulation of Wnt pathway genes and TNKS overexpression in human and murine lung cancer, we next asked whether inhibition of the Wnt pathway with the TNKS inhibitors XAV939, IWR-1 endo, or IWR-1 exo would inhibit growth of murine or human lung cancer cell lines. Individual treatments with increasing doses of these three compounds (100nM to 50 μM) dose-dependently decreased proliferation of the ED1, ED1L, and ED2 murine lung cancer cell lines in comparison to vehicle controls after 3 days of treatment (Figure 2A with statistical analysis in Additional file 2: Figure S2). Independent treatments of the human lung cancer cell lines A549, Hop62, and H522 dose-dependently decreased proliferation of each of these lines versus vehicle controls after 3 days of culture (Figure 2B with statistical analysis in Additional file 2: Figure S2). The compounds also inhibited growth of the C10 murine immortalized bronchial epithelial cell line (Figure 2C, left panel). The BEAS-2B immortalized human bronchial epithelial cell line was found to be more resistant to growth inhibition by these agents, except for the IWR-1 endo compound (Figure 2C, right panel). Additionally, treatments of the ED1 cell line plated under colony-forming conditions with 10 μM of each TNKS inhibitor significantly inhibited 7-day colony formation relative to vehicle control, as shown with colony number quantification (Figure 2D).

Cells were growth inhibited by these compounds but did not show appreciable apoptosis (data not shown). In drug washout experiments, normal cell growth was restored after removal of the drugs, confirming that the effects of these compounds are reversible (Additional file 3: Figure S3).

### TNKS inhibitors act as canonical Wnt pathway inhibitors in lung cancer

**Figure 3:**
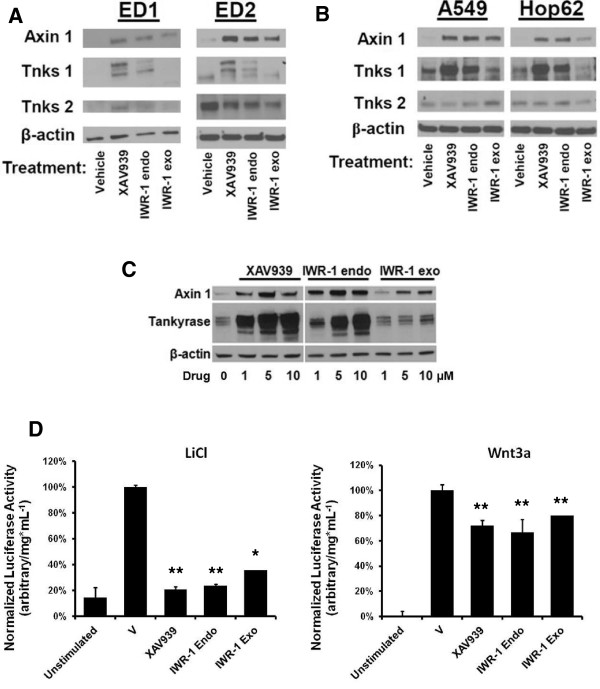
**TNKS inhibition antagonizes canonical Wnt signaling in lung cancer.** (**A**) Immunoblots for Wnt pathway components axin 1, TNKS1, and TNKS2 are shown in ED1 (left panel) and ED2 (right panel) murine lung cancer cell lines following 3 days treatment with TNKS inhibitors or vehicle. (**B**) Immunoblots are shown for Wnt pathway components, as in panel A, in A549 (left panel) and Hop62 (right panel) human NSCLC cell lines following 3 days treatment with TNKS inhibitors or vehicle. (**C**) Dose-response of Wnt pathway component stabilization is shown in ED1L cells following 3 days treatment with TNKS inhibitors or vehicle at doses shown. (**D**) Activity of a lentiviral Wnt-responsive luciferase construct stably expressed in the ED1 cell line was measured following 16 hours cotreatment with TNKS inhibitors and a Wnt activator: 20 mM LiCl (left panel) or 25 ng/mL recombinant murine Wnt3a (right panel). Luciferase activity was normalized to total protein concentrations in each sample and compared to vehicle control. Error bars represent SD of three experiments in triplicate. (* *p* ≤ 0.05).

We sought to validate whether the TNKS inhibitors exerted effects on Wnt signaling in human and murine lung cancer cell lines. Immunoblot analyses of ED1 and ED2 murine lung cancer cell lines (Figure 3A) and A549 and Hop62 human lung cancer cell lines (Figure 3B) following treatment with 10 μM of each inhibitor revealed stabilization of TNKS1, as expected due to inhibition of TNKS auto-PARsylation function [45]. Stabilization of TNKS2 was only apparent in ED1. We also observed accumulation of axin 1 at the protein level in all cell lines, a key component of the axin/APC/GSK3 β-catenin destruction complex and direct target of TNKS PARsylation. This relationship appeared to be dose-dependent, as evidenced in the ED1L cell line following 3 days of treatment with the inhibitors at increasing doses (Figure 3C).

Stable infection of ED1L (data not shown) or ED1 (Figure 3D) with a lentiviral vector containing a luciferase gene cassette under control of a 7× TCF binding site promoter [36] allowed for monitoring of the transcriptional activity of the Wnt pathway during TNKS inhibition. Wnt reporter activity was normalized to the total protein content of the cells to account for growth inhibitory effects. Basal (unstimulated) activity of the Wnt reporter was low in both cell lines, and was not significantly affected by tankyrases inhibition (data not shown). Co-treatment of either cell line with a Wnt activator (20 mM LiCl or 25 ng/mL recombinant murine Wnt3a) and a 10 μM dosage of each TNKS inhibitor led to a significant reduction in normalized Wnt-responsive luciferase activity versus vehicle controls at 16 hours post-stimulation (Figure 3D).

### siRNA-mediated repression of TNKS1 and TNKS2 in cancer cell lines

**Figure 4:**
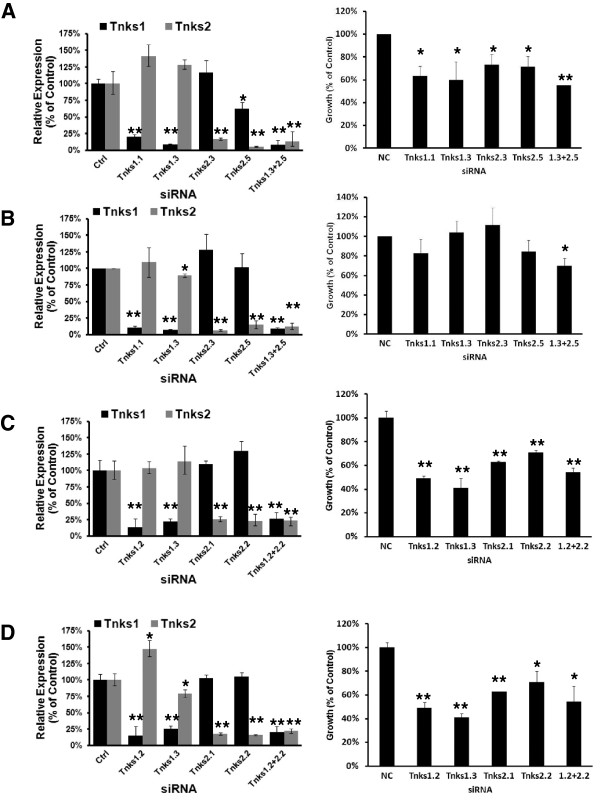
**Transient TNKS knockdown in lung cancer cell lines.** Antineoplastic consequences of specific genetic knockdown of the TNKS via siRNAs are shown. Two independent siRNAs targeting respectively each of TNKS1 or TNKS2, or a non-targeting control were transfected into (**A**) ED1, (**B**) ED2, (**C**) A549, and (**D**) Hop62 cells, alone or in combination. Gene expression levels of TNKS1 and TNKS2, measured by qPCR assays, are shown following knockdown for 24 hours (left panels) as compared to control. Proliferative consequences were measured by luminescent cell viability assay after 3 days culture (right panels). Error bars represent SD of three experiments in triplicate. (* *p* ≤ 0.05, ** *p* ≤ 0.01).

Because tool compounds such as XAV939 and IWR-1 likely have off-target effects, we sought to validate that the antineoplastic activity of these inhibitors was due to specific TNKS antagonism through independent genetic approaches. Transient TNKS knockdowns were accomplished with two siRNAs that independently target TNKS1 or TNKS2 in human or mouse cells. Transfection of these siRNAs alone or in combination led to significant (*P* ≤ 0.05) repression of TNKS1 or TNKS2 at the mRNA level in ED1 (Figure 4A, left panel) and ED2 (Figure 4B, left panel) murine cell lines at 24 hours, versus controls. Similar TNKS knockdown was achieved in the human lung cancer cell lines A549 (Figure 4C, left panel) and Hop62 (Figure 4D, left panel).

**Figure 5:**
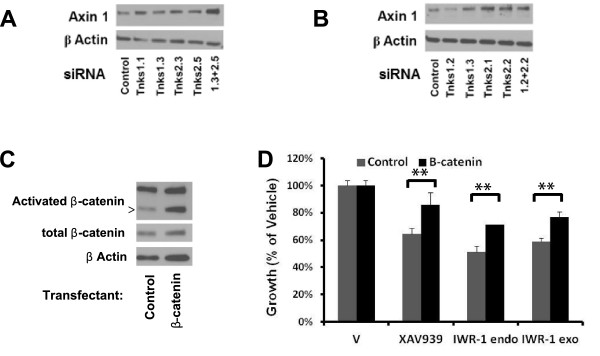
***In vitro *effects of TNKS knockdown and rescue of Wnt pathway inhibition.** (**A**) Protein expression levels of axin 1 following transient TNKS knockdown are shown by immunoblot analysis in ED1. (**B**) Protein expression levels of axin 1 following transient TNKS knockdown are shown by immunoblot analysis in Hop62. (**C**) Expression of activated β-catenin and total β-catenin in ED1 cells following stable infection with constitutively active β-catenin or empty vector. (**D**) Growth inhibition of ED1 cells infected as in C by TNKS inhibitors for 3 days at 10 μM is shown by luminescent cell viability assay. Error bars represent SD of three experiments in triplicate. (* *p* ≤ 0.05, ** *p* ≤ 0.01).

The consequences of TNKS knockdown on proliferation for each of the examined NSCLC cell lines was assessed after 3 days culture versus non-targeting control siRNA. Single knockdown of either TNKS1 or TNKS2 or dual TNKS knockdowns were growth inhibitory in the ED1, A549, and Hop62 lung cancer cell lines (Figures 4A, 4C, and 4D, respectively). In ED2 cells, only the combined knockdown achieved significant growth repression versus control (Figure 4B). The dual siRNA knockdown of TNKS1 and TNKS2 was not significantly more growth-suppressive than was either of the individual knockdowns in ED1, A549, or Hop62 cells. The growth inhibitory effects of TNKS knockdown were accompanied by stabilization of axin1 at the protein level, as shown in ED1 (Figure 5A) and Hop62 cells (Figure 5B).

### Rescue of TNKS inhibitor growth effects by activated β-catenin

To further show Wnt pathway specificity of *in vitro* antineoplastic effects of these TNKS inhibitors, we performed a rescue experiment. ED1 cells were stably infected with an empty vector or an activated form of β-catenin that cannot be phosphorylated by GSK3 and thus remains constitutively active. Expression of this construct was confirmed at the protein level (Figure 5C). Individual treatments of these stable cell lines with 10 μM TNKS inhibitors for 3 days showed differential growth inhibition between β-catenin expressing cells and controls, with a significant rescue of ~20% growth in each case (Figure 5D).

### shRNA repression of TNKS1 and TNKS2 has antineoplastic activity

**Figure 6:**
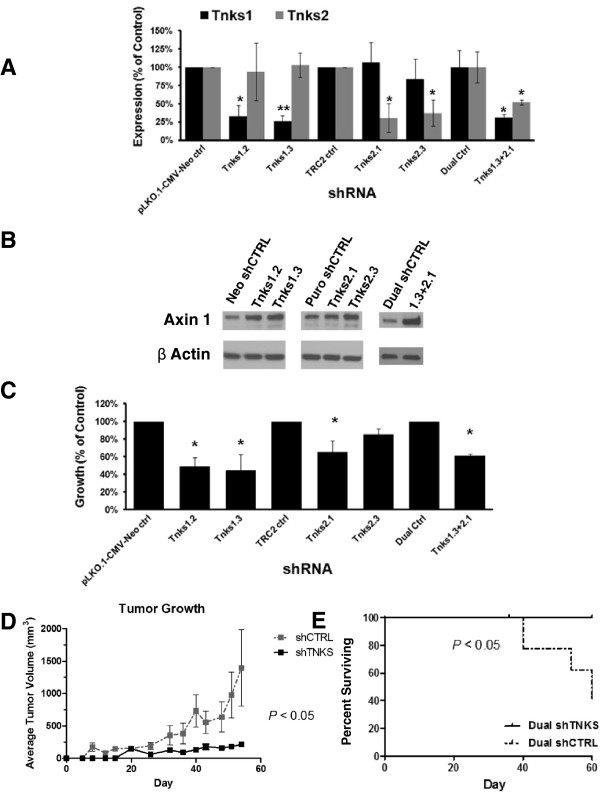
***In vivo *consequences of TNKS knockdown.** (**A**) ED1 cells were infected with lentiviral shRNA constructs targeting TNKS1 or TNKS2 at two independent sites each, or the combination, and were selected with G418 (TNKS1 constructs and pLKO.1-CMV-Neo control), puromycin (TNKS2 constructs and TRC2 control), or the combination (dual control and dual shRNA). mRNA expression levels of the indicated species are detected by qPCR. (**B**) Axin 1 protein levels following stable TNKS knockdown are shown by immunoblot analysis. (**C**) Consequences on proliferation of stable TNKS knockdown in ED1 cells are shown i*n vitro* as measured by luminescent cell viability assay 3 days after plating. (**D**) ED1 TNKS1 and TNKS2 shRNA dual transductants (or control) were injected into the flanks of athymic nude mice and tumor diameters measured twice weekly. Tumor growth rates are shown, *N* = 10 in each arm +/- SEM. (**E**) Time to the specified endpoint (designated as percent surviving) for the xenograft study is shown. Error bars represent SD of the mean. (* *p* ≤ 0.05).

To confirm independently the transient *in vitro* knockdown results in the *in vivo* setting, we achieved stable genetic knockdown of the TNKS, alone or in combination, in the ED1 murine lung cancer cell line with the indicated TNKS shRNAs versus control shRNAs (Figure 6A). ED1 cells were infected with independent shRNAs targeting TNKS1 or TNKS2, or both species. Stable knockdown at the mRNA level was achieved following G418 (TNKS1 and pLKO.1-cmv-neo scrambled control) or puromycin (TNKS2 and TRC2 control) selection, or selection for both shRNA transductants in the dual knockdown study. Knockdown of TNKS1 caused an accumulation of axin 1, as did independent knockdown of TNKS2, although to a lesser extent; combined TNKS knockdown showed increased stabilization of axin 1 (Figure 6B). The *in vitro* growth inhibitory effects of stable TNKS knockdown are shown (Figure 6C) and are similar to those observed from siRNA-mediated transient TNKS repression.

ED1 murine lung cancer cells after dual TNKS1 and TNKS2 stable knockdown were selected for xenograft studies in nude mice or tail vein injection into syngeneic FVB mice. Mice bearing xenografts of the TNKS knockdown cells showed a decrease in tumor growth rate (Figure 6D) and increase in time to the specified endpoint (Figure 6E) as compared to controls. As expected from the *in vitro* results, shRNA-mediated knockdown of TNKS1 and TNKS2 led to a significant decrease in syngeneic tumor formation after 8 weeks (Additional file 4: Figure S4).

## Discussion

Aberrant Wnt signaling has long been associated with carcinogenesis. Both familial and sporadic colorectal cancers were among the first to be associated with the Wnt pathway, as a large percentage of these cases harbor driver mutations in *APC* or *β-catenin*[13-15,46,47]. Subsequently, deregulation or mutation of components of the canonical and non-canonical arms of the Wnt pathway were linked to hematopoietic cancers such as acute myelogenous leukemia (AML) [48] and solid tumors including breast cancer [49,50], ovarian cancer [51], and NSCLC [52], among other malignancies. The results of our microarray studies of cyclin-E driven murine lung adenocarcinomas revealed deregulation of specific components of the Wnt pathway, both canonical and noncanonical, in agreement with prior reports [7,16-19].

TNKS1 and/or TNKS2 levels were elevated in the majority of the paired tumor and normal murine transgenic cyclin E samples. In the three evaluable human lung adenocarcinoma and normal lung pairs TNKS1 and TNKS2 levels were either moderately elevated or unchanged. There is a paucity of literature on differential TNKS expression at either the mRNA or the protein level in cancer. In addition to potential regulation at the mRNA level, however, the TNKS are known to be regulated post-transcriptionally. The RING-type E3 ubiquitin ligase RNF146 has been identified as a PAR-dependent E3 enzyme that mediates ubiquitylation of both axin and the TNKS themselves [53,54]. RNF146 is found in a breast cancer susceptibility locus at *6q22.33*, with overexpression of the locus [55] but not mutation [56] correlated with increased breast cancer risk in both Ashkenazi Jewish and non-Jewish women. Deregulation of post-transcriptional TNKS regulators cannot be accounted for in our analysis, and future studies are planned to assess the association between tumorigenicity and TNKS expression at both mRNA and protein levels across a broader sample set.

We and others have hypothesized [6] that pharmacological targeting of the Wnt pathway would treat or even prevent some malignancies, including lung cancer, where survival remains poor despite current treatments [1]. The development of Wnt pathway pharmacological inhibitors has proven to be a challenge. The large protein-protein interaction domains responsible for signal transduction at the level of the β-catenin destruction complex and β-catenin/TCF/Lef interactions make it difficult to target these components with small molecules. Some progress has been made designing compounds targeting these interactions, but those compounds have not yet shown *in vivo* efficacy, as reviewed [57]. Hence, the discovery of the TNKS as activating enzymes in the Wnt pathway [58] and the development of tool compounds inhibiting their activity [20,21] were each positive steps towards small-molecule Wnt pathway inhibition. Our results with the first generation of TNKS inhibitors, XAV939 and the IWR-1 compounds, indicate that they have antiproliferative effects in lung cancer cell lines.

Each cell line examined exhibited a distinct response profile for each of the three TNKS inhibitors. This likely resulted from a differing reliance of each on active Wnt signaling *in vitro*. In most, but not all cases, the IWR-1 exo enantiomer was less growth inhibitory than was the IWR-1 endo enantiomer. This was expected from the difference in EC_50_ between the compounds [21]. These effects were found in standard serum concentration culture conditions. In a recent study in breast cancer cell lines, growth inhibition by XAV939 was only seen under conditions of reduced serum [59]. We are currently examining the effects of different growth conditions on TNKS inhibitor activity in our lung cancer models. The lack of effect on apoptosis suggests that the inhibition of proliferation was due to growth arrest or through a mechanism other than programmed cell death. As expected from the noncovalent nature of TNKS inhibition by XAV939 and the IWR-1 isomers as determined in structure-activity relationship studies [25,60-62], the growth inhibitory effects of all three compounds washed out fully.

Despite being closely-related cell lines derived from adenocarcinomas of mice differing only in the proteasomal susceptibility of their human cyclin E transgene [31,39], the molecular and growth phenotypic responses of the ED1 and ED2 cell lines to TNKS inhibition differed. Specifically, the latter failed to accumulate TNKS2 following inhibitor treatment and was only growth inhibited by combined TNKS knockdown. This may speak to stochastic differences in post-translational regulation of TNKS enzymes, potentially a result of the specific niche or inflammatory milieu in which the original tumors developed in their respective animals.

Although the BEAS-2B human immortalized bronchial epithelial cell line was relatively resistant to TNKS inhibitors, the ability of TNKS inhibition to growth inhibit the murine C10 cell line raises concerns regarding therapeutic window and toxicity profiles. Although the original reports which described the IWR-1 isoforms and XAV399 included *in vivo* inhibition of Wnt-mediated tailfin regeneration in zebrafish [20,21], to our knowledge only a single additional study has used XAV939 successfully *in vivo*[63]. Further development of TNKS inhibitors for *in vivo* use has recently shown promise [64,65]. In the former study, no overt toxicities were reported; however, evidence of colon crypt toxicity was observed in the latter at high doses. Whether a sufficient therapeutic window exists between TNKS inhibition in cancer cells and normal cells is still an open question, as is the toxicity profile of the class.

We provide evidence here that the antineoplastic effects of TNKS antagonists are through inhibition of the Wnt pathway and are not solely due to off-target effects of these inhibitors. Although the TNKS are key regulators of canonical Wnt signaling, they are known to have other effects. These include the maintenance of telomeres and activation of telomerase through binding and PARsylation of TRF1 [66,67], and directing proper polymerization of mitotic spindles through PARsylation of NuMA [23,24]. Our results do not rule out effects of TNKS inhibition acting in part through these or other potential mechanisms. However, disruption of cancer immortalization by inhibition of telomere extension would exert additional antineoplastic effects. We have recently reported that targeting chromosome stability in cancer cells is also an antineoplastic target [40]. In fact, the inability of constitutively activated exogenous β-catenin to fully rescue growth inhibition due to TNKS inhibition in ED1 cells may speak to TNKS action in other pathways, or to off-target effects of the tool compounds themselves. It is also possible that insufficient exogenous expression was achieved to stoichiometrically out-compete all of the available destruction complex in the face of TNKS inhibition.

Our results show *in vivo* anti-cancer effects of TNKS knockdown. Combined with the *in vitro* results with the described inhibitors, this suggests a potential for clinical benefit from TNKS inhibition in lung cancer. A clear limitation of these findings is that xenograft studies in nude mice do not fully recapitulate the tumorigenic milieu of the lung, although the pilot syngeneic study presented here begins to speak to *in vivo* relevance in the tumor microenvironment. In addition, genetic knockdown of enzymes is likely to have effects distinct from pharmacological inhibition due to the alteration of protein number rather than inhibition of enzymatic activity. To address both of these points, future studies will treat cyclin E overexpressing mice [31] with next-generation TNKS inhibitors in both chemopreventative and chemotherapeutic modalities to assess their benefit as single agents or in combination to prevent or treat lung cancer.

The Wnt pathway is known to contribute to lung cancer progression [8,68] and also to metastasis [9]. In addition, the Wnt pathway is important in the maintenance and self-renewal of stem cell compartments, and has been linked to the growth of cancer stem cell populations of breast [69] and lung [70] cancers. Thus, antagonizing the Wnt pathway through TNKS inhibition may serve to overcome drug resistance in the cancer stem cell niche and thereby reduce outgrowth of these intrinsically drug-resistant cells.

A recent publication confirmed key aspects of the hypothesis presented here [71]. Distinct from our candidate-gene approach, the authors pursued an shRNA-based screen for synergistic interactions with EGFR inhibition and uncovered a similar role for TNKS inhibitors in NSCLC. However, the authors saw very little *in vivo* antineoplastic effects from *TNKS1* knockdown alone, in contrast to the significant growth effects we observed following *TNKS1* and *TNKS2* combined knockdown. We propose that this discrepancy is likely due to the ability of TNKS2 to compensate for TNKS1 in long-term knockdown, as is seen in *in vivo* xenograft studies lasting upwards of 60 days. In addition, the present work sheds additional light on the actions of the TNKS inhibitors as single agents and conclusively shows growth inhibitory effects through inhibition of the canonical Wnt pathway.

## Conclusions

We have shown here that specific components of the Wnt signaling pathway are deregulated in a murine transgenic cyclin E model of lung adenocarcinoma and in human lung cancer. Pharmacological or genetic inhibition of TNKS1 and TNKS2 antagonizes canonical Wnt signaling and reduces lung cancer proliferation *in vitro* and *in vivo*. Our findings provide evidence for TNKS1 and TNKS2 as antineoplastic targets in NSCLC. Further studies of these targets and development of small molecule inhibitors for clinical testing in lung cancer are warranted.

## Abbreviations

ABC: Activated β-catenin; AML: Acute myelogenous leukemia; ANOVA: Analysis of variance; APC: Adenomatous polyposis coli; BSA: Bovine serum albumin; Dvl: Disheveled; FBS: Fetal bovine serum; Fzd: Frizzled; GSK3: Glycogen synthase kinase-3; IACUC: Institutional Animal Care and Use Committee; IRB: Institutional review board; LEF: Lymphoid-enhancer binding factor; NSCLC: Non-small cell lung cancer; PAR: Poly-ADP-ribose; PARP: Poly-ADP-ribose polymerase; PBS: Phosphate buffered saline; qPCR: Semi-quantitative real time polymerase chain reaction; RMA: Robust multichip average; SD: Standard deviation; SDS-PAGE: Sodium dodecyl sulfate polyacrylamide gel electrophoresis; SEM: Standard error of the mean; siRNA(s): Small interfering ribonucleic acids; shRNA(s): Small hairpin ribonucleic acids; SP-C: Surfactant protein C; TBST: 0.1% Tween-20 tris buffered saline; TCF: T-cell factor; TNKS1: TRF1-interacting, ankyrin-related ADP-ribose polymerase, Tankyrase 1; TNKS2: TRF1-interacting, ankyrin-related ADP-ribose polymerase 2, Tankyrase 2; Wif-1: Wnt inhibitory factor 1.

## Competing interests

The authors declare that they have no competing interests.

## Authors’ contributions

AMB participated in the design of the study and performed molecular and cellular biology and *in vivo* studies, interpreted the results, prepared the figures, and wrote the manuscript. KCJ performed molecular and cellular biology experiments and assisted in interpreting the results. RVS performed pathological analysis. AS assisted with *in vivo* studies. YA contributed to the overall scientific direction of the study. ED contributed to the overall scientific direction, experimental design and interpretation, and in manuscript and figure preparations. SJF directed the overall study and the preparation of the manuscript. All authors read and approved the final manuscript.

## Pre-publication history

The pre-publication history for this paper can be accessed here:

http://www.biomedcentral.com/1471-2407/13/211/prepub

## Supplementary Material

Additional file 1: Figure S1 Table containing all antibodies and dilutions, sequences for primers used to measure gene expression levels by qPCR, and siRNA and shRNA sequences used to knock-down TNKS expression levels. Click here for file

Additional file 2: Figure S2 Table reporting statistical analysis of main body figure 2 panels A, B, and C. Percent growth at each drug dosage was compared to vehicle control via ANOVA with Dunnett’s multiple comparisons post-test. Click here for file

Additional file 3: Figure S3 Figure showing washout studies for ED1 and A549 lung cancer cell lines treated with TNKS inhibitors. Click here for file

Additional file 4: Figure S4 *In vivo* syngeneic lung cancer tumor formation from injection of FVB mice with ED1 cells transduced with dual TNKS knockdown or dual control. Click here for file
