# Supplementary material for: Evidence for tankyrases as antineoplastic targets in lung cancer
Source: BMC Cancer. 2013 Apr 28;13:211. doi: 10.1186/1471-2407-13-211 (PMC3644501; doi:10.1186/1471-2407-13-211)
Supplement: Additional file 1: Figure S1 — Table containing all antibodies and dilutions, sequences for primers used to measure gene expression levels by qPCR, and siRNA and shRNA sequences used to knock-down TNKS expression levels. [file 1471-2407-13-211-S1.pdf]

Supplemental Figure 1

Antibodies Used in These Analyses

Antibody Name	Supplier	Catalog #	Dilution	Notes
actin C-11	Santa Cruz	SC-1615	1:2000	
activated β-catenin (ABC)	Millipore	05-665	1:500	Diluted in 1% milk in TBST
axin 1	Cell Signaling	C76H11	1:1000	
β-catenin	BD Biosciences	610153	1:2000	
Tankyrase	AbCam	ab13587	1:500	Used to detect murine Tnks1
Tankyrase 1	Santa Cruz	SC-8708-R	1:1000	Used to detect human Tnks1
Tankyrase 2	Everest Biotech	EB08220	1:1000	

Real-Time PCR Primers Used in These Analyses

Name	Species	Forward Primer (5'-3')	Reverse Primer (5'-3')
β-actin	human	GCTACGAGCTGCCTGACGG	GATGGAGTTGAAGGTAGTTTCG
GAPDH	human	ATGGGGAAGGTGAAGGTCG	GGGGTCATTGATGGCAACAATA
Tnks1	human	GCAGTACCACCAGCACAATC	AGGGGAGGATGGAGAGGAAG
Tnks2	human	ATCTGCTCTGCCCTCTTGTTACAA	GCTAAAATCTACTCCTGGAACCTC
Wif1	human	GGTTCCACGGACCTCACTGT	AGTCACACAAAGTCCACCATTCA
β-actin	mouse	TTGTCCCCCCAACTTGATGT	GCCCTGGCTGCCTCAAC
GAPDH	mouse	AGGTCGGTGTGAACGGATTTG	TGTAGACCATGTAGTTGAGGTCA
Tnks1	mouse	GTCTACTCCGTTACACCTGGC	TGAAGAGGTACAAGTCCACCTTT
Tnks2	mouse	CGCCCGAGAAGGTGAACAG	TTTGCACCGTTCTGAAGAAGAT
Wif1	mouse	GATTTCAGGAAAGCCCAACAAAGAA	GTTGGATCTGCCATGATGCCTTT

siRNAs Used in These Analyses

Name	Gene Target	Species	Duplex Forward Oligo (5'-3')	Duplex Reverse Oligo (5'-3')
Control	scrambled	human + mouse	CGUUAAUCGCGUAUAAUACGCGUAT	AUACGCGUAUUAUACGCGAUUAACGAC
Tnks1.1	Tnks1	mouse	GGAUGUUGUAGAACACUUGCUGCAG	CUGCAGCAAGUGUUCUACAACAUCCUU
Tnks1.3	Tnks1	mouse	GCACGGAGCAAAGAUGAAUGCACTG	CAGUGCAUUCAUCUUUGCUCCGUGCUU
Tnks2.3	Tnks2	mouse	AGGUUAAUGCUUUGGAUAGUCUUGG	CCAAGACUAUCCAAAGCAUUAACCUUU
Tnks2.5	Tnks2	mouse	CGGAUGUCCAAUUCACAAAGACAGA	UCUGUCUUUGUGAAUUGGACAUCCGGU
Tnks1.2	Tnks1	human	GGCAAGCAAAUAAUGUAGAUUAACA	UGUUAAUCUACAUUAUUUGCUUGCCAU
Tnks1.3	Tnks1	human	GCAUGUUAAAGUCCUCUAGUCAUCA	UGAUGACUAGAGGACUUUAACAUGCUG
Tnks2.1	Tnks2	human	AGCUCAUAAUGAUGUUGUUGAAGTA	UACUUCAACAACAUCAUUAUGAGCUUU
Tnks2.2	Tnks2	human	GGAACAUAAGUAGGAUGUUACAUTT	AAAUGUAACAUCCUACUUAUGUUCCAU

shRNAs Used in These Analyses

Name	Gene Target	Backbone	TRC Identifier	Notes
pLKO.1-CMV-Neo shCTRL	scrambled	pLKO.1-CMV-Neo (G418 selectable)	n/a	Scrambled sequence from SHC202 custom-cloned by Sigma Aldrich
Tnks1.2	murine Tnks1	pLKO.1-CMV-Neo (G418 selectable)	TRCN0000238900	custom-cloned by Sigma Aldrich
Tnks1.3	murine Tnks1	pLKO.1-CMV-Neo (G418 selectable)	TRCN0000238901	custom-cloned by Sigma Aldrich
TRC2 shCTRL	scrambled	TRC2 (puromycin selectable)	n/a	Sigma product SHC202
Tnks2.1	murine Tnks2	TRC2 (puromycin selectable)	TRCN0000111416	
Tnks2.3	murine Tnks2	TRC2 (puromycin selectable)	TRCN0000111418	
