# Supplementary material for: Evidence for tankyrases as antineoplastic targets in lung cancer
Source: BMC Cancer. 2013 Apr 28;13:211. doi: 10.1186/1471-2407-13-211 (PMC3644501; doi:10.1186/1471-2407-13-211)
Supplement: Additional file 2: Figure S2 — Table reporting statistical analysis of main body figure 2 panels A, B, and C. Percent growth at each drug dosage was compared to vehicle control via ANOVA with Dunnett’s multiple comparisons post-test. [file 1471-2407-13-211-S2.pdf]

Supplemental Figure 2

	ED1			ED1L			ED2		
	IWR-1 Exo	IWR-1 Endo	XAV 939	IWR-1 Exo	IWR-1 Endo	XAV 939	IWR-1 Exo	IWR-1 Endo	XAV 939
100nM	n.s.	n.s.	n.s.	n.s.	n.s.	n.s.	n.s.	**	*
500nM	n.s.	**	**	n.s.	n.s.	n.s.	n.s.	**	**
1µM	n.s.	**	**	n.s.	*	n.s.	n.s.	**	**
5µM	**	**	**	**	**	n.s.	**	**	**
10µM	**	**	**	**	**	*	**	**	**
25µM	**	**	**	**	**	**	**	**	**
50µM	**	**	**	**	**	**	**	**	**

	A549			Hop62			H522		
	IWR-1 Exo	IWR-1 Endo	XAV 939	IWR-1 Exo	IWR-1 Endo	XAV 939	IWR-1 Exo	IWR-1 Endo	XAV 939
100nM	n.s.	n.s.	n.s.	n.s.	n.s.	n.s.	n.s.	n.s.	n.s.
500nM	n.s.	n.s.	*	n.s.	n.s.	n.s.	n.s.	n.s.	n.s.
1µM	n.s.	**	**	n.s.	**	n.s.	n.s.	**	n.s.
5µM	n.s.	**	**	n.s.	**	**	n.s.	**	*
10µM	**	**	**	n.s.	**	**	n.s.	**	**
25µM	**	**	**	n.s.	**	**	n.s.	**	**
50µM	**	**	**	**	**	**	**	**	**

	C10			BEAS2B		
	IWR-1 Exo	IWR-1 Endo	XAV 939	IWR-1 Exo	IWR-1 Endo	XAV 939
100nM	n.s.	*	n.s.	n.s.	n.s.	n.s.
500nM	n.s.	**	**	n.s.	n.s.	n.s.
1µM	**	**	**	n.s.	n.s.	n.s.
5µM	**	**	**	*	**	*
10µM	**	**	**	*	**	*
25µM	**	**	**	n.s.	**	**
50µM	**	**	**	**	**	**

Supplemental Figure 2: Statistical analysis of figure 2 panels A, B, and C. Percent growth at each drug dosage was compared to vehicle control via ANOVA with Dunnett's multiple comparisons post-test in GraphPad InStat. (* $p < 0.05$, ** $p < 0.01$)
