# Supplementary material for: Evidence for tankyrases as antineoplastic targets in lung cancer
Source: BMC Cancer. 2013 Apr 28;13:211. doi: 10.1186/1471-2407-13-211 (PMC3644501; doi:10.1186/1471-2407-13-211)
Supplement: Additional file 3: Figure S3 — Figure showing washout studies for ED1 and A549 lung cancer cell lines treated with TNKS inhibitors. [file 1471-2407-13-211-S3.pdf]

Supplemental Figure 3

A

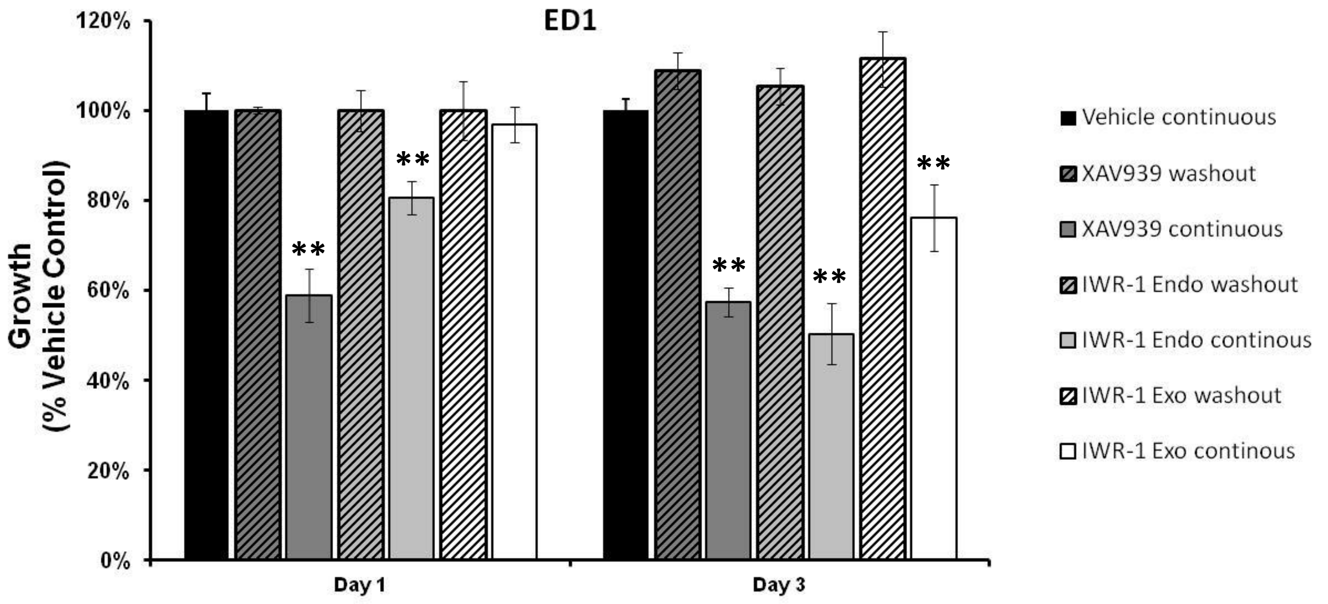

B

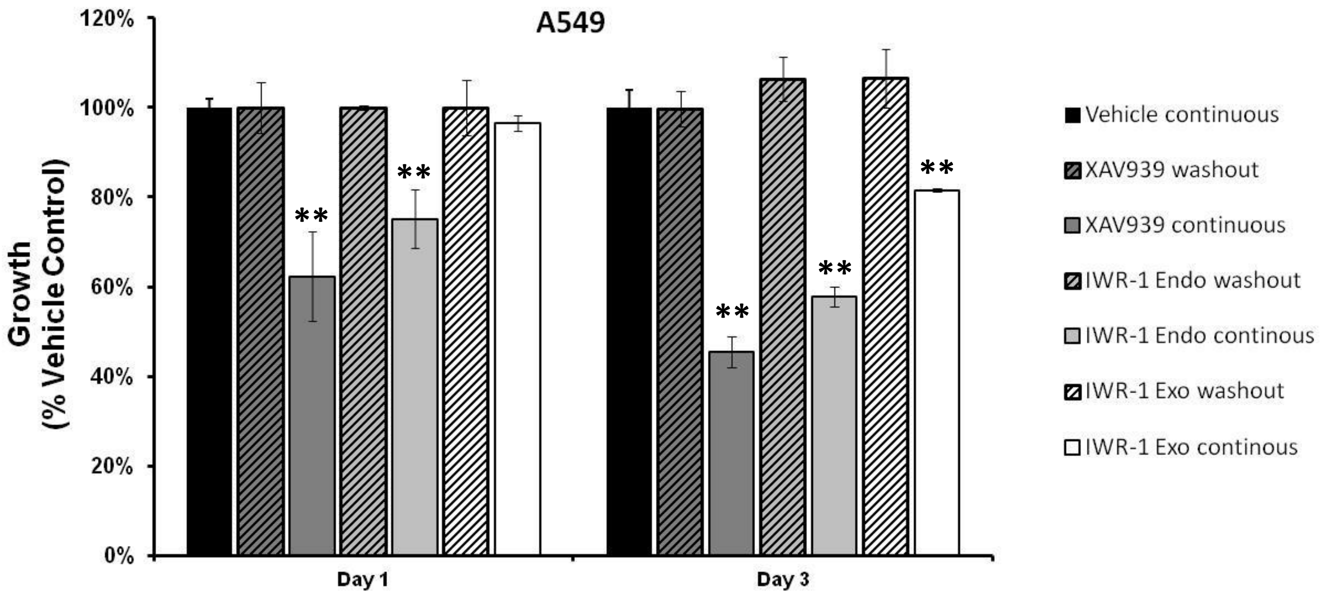

Supplemental Figure 3

(a) ED1 and (b) A549 cells were treated for 3 days with vehicle or Tnks inhibitor (10μM). This led to growth inhibition as seen in figure 2 (data not shown). Cells were then trypsinized and replated at equal cell density for another 3 day treatment with either the respective drug (continuous treatment) or washout. Cell growth was measured at days 1 and 3 by luminescent cell viability assay. Error bars represent mean +/- S.D. of two experiments in triplicate. Comparisons were made between each treatment or washout group and the continuous vehicle treatment control. (** $P < 0.01$)
