# Supplementary material for: Evidence for tankyrases as antineoplastic targets in lung cancer
Source: BMC Cancer. 2013 Apr 28;13:211. doi: 10.1186/1471-2407-13-211 (PMC3644501; doi:10.1186/1471-2407-13-211)
Supplement: Additional file 4: Figure S4 — In vivo syngeneic lung cancer tumor formation from injection of FVB mice with ED1 cells transduced with dual TNKS knockdown or dual control. [file 1471-2407-13-211-S4.pdf]

Supplemental Figure 4

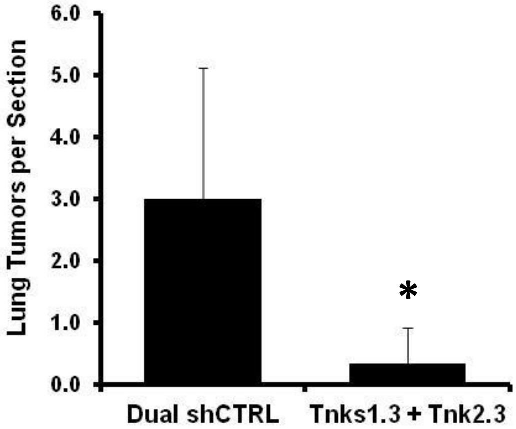

Supplemental Figure 4

In vivo tumor formation from injection of syngeneic FVB mice with ED1 cells transduced with dual TNKS knockdown or dual control, *N* = 5 and 3, respectively. Error bars represent mean +/- S.D. (* $p < 0.05$ by one-tailed T-test)
